# Mr. Montgomery on the Contagious Nature of Cholera Morbus

**Published:** 1826-07

**Authors:** 


### SOME CASES OF CHOLERA MORBUS.*

About the middle of May, 1825, a convict was sent from a village, where cholera had appeared, to Chanda, eighty miles to the westward. Two hours after his arrival at this place, he was attacked with the epidemic, and in two hours more Mr. Montgomery saw him. His symptoms were—vomiting and purging of a watery fluid; severe spasms of the abdominal and gastrocnemii muscles; thirst; coldness of the extremities: clammy perspirations: anxious countenance: jactitation: great prostration of strength. Laudanum, oil of mint, brandy, the usual treatment in that part of India were employed, but in twelve hours from the first attack, he was dead. He was carried out for burial by four other convicts: three of them were attacked on that and the next day with similar symptoms, and two died in eighteen hours. A native who gave them their medicines, was seized with cholera on his return from the jail, and nearly perished. The disease now spread among the convicts, (those only who had been in communication with the persons who laboured under it suffered) and six died in as many days.

Further; four police-men escorting treasure from a distant village to Chanda passed a night at Mhool, where the disease was then raging—the next day two of them were taken ill on the road and died in six hours. The other two, a few hours after their arrival in Chanda, were seized with cholera and died likewise within six hours.

Finding his practice unsuccessful, indeed inert, Mr. M. bethought himself of the following—a pill of two grains of opium, ten of calomel, and three of powdered capsicum, together with a draught of two ounces of aqua vitæ, fifty drops of laudanum and ten of oil of peppermint was

* Mr. Montgomery, Repository for May, 1826.

given as soon as possible after the appearance of the disease. If the vomiting and purging continued, the draught was repeated every half hour and the pill every fourth hour : if this was rejected it was repeated till it was retained. The warm bath, blisters to the epigastric region and frictions with hot arrack, to the trunk and extremities, were also used—bleeding was not employed, our author's practice being solely among the natives who will not bear depletion. Under this treatment, 50 out of 62 recovered, no case proving fatal in which bile was ejected by vomiting or otherwise. Large quantities of the pills and mixture were distributed amongst the natives, with the happiest effect; the rate of mortality from the epidemic being reduced from 8 out of 10 to one in 15.

If the above relation be perfectly correct, it certainly affords strong presumptive evidence in favour of the contagious nature of cholera morbus. But when we consider how many errors human judgment is liable to, and how often coincidences are set down for consequences, we must say the paper does not carry perfect conviction to our minds.

